# Monarch butterfly conservation through male germplasm cryopreservation

**DOI:** 10.1038/s41598-024-65624-x

**Published:** 2024-06-28

**Authors:** Courtney C. Grula, Arun Rajamohan, Joseph P. Rinehart

**Affiliations:** Insect Genetics and Biochemistry Edward T. Schafer Research Center, U.S. Department of Agriculture/Agricultural Research Center, 1616 Albrecht Boulevard, Fargo, ND 58102 USA

**Keywords:** Entomology, Zoology, Conservation biology

## Abstract

Monarch butterfly (*Danaus plexippus* L.) populations have declined in North America. The International Union for Conservation of Nature (IUCN) recently classified the species as endangered, sparking public concern and conservation efforts. Our approach to conservation is through cryopreservation of germinal cells and tissue. The goal of this study was to develop a cryopreservation protocol for monarch spermatozoa to ensure successful long-term storage. Cryopreserved sperm cells would provide a reserve of monarch germplasm, which could be utilized in the event of population loss. In this study, sperm cell bundles collected from male monarch butterflies were cryopreserved in a cryoprotective medium and stored in liquid nitrogen. To determine the post-cryopreservation sperm cell viability, a subsample of preserved sperm bundles were thawed rapidly, and their viability was qualified using a sperm live/dead stain. We are presenting a protocol to preserve and store genetic material and viable sperm bundles of the monarch butterfly. To date, this is the first report of successful cryopreservation of monarch germplasm which sets the foundation for cryostorage and could be extensible to other vulnerable lepidopterans.

## Introduction

Populations of historically abundant insects are declining, which has generated concern for catastrophic loss of biodiversity^1^. Conspicuous and charismatic, Lepidopterans (butterflies and moths) are sentinel insects with documented long-term declines in many species^2–5^. In particular, the population of monarch butterflies (*Danaus plexippus* L*.*), which are important in providing pollination services for a number of wild flora and also has cultural significance^6^, have declined over 80% in the last decade^7,8^. The International Union for the Conservation of Nature (IUCN)^9^ recently classified the monarch as endangered, and the US Fish and Wildlife Service has petitioned for the monarch to be considered as threatened under the Endangered Species Act^10^. Although, more recently, monarchs have been re-classified by the IUCN as vulnerable to extinction^11^.

To save any species, conservation efforts must preserve genetic diversity as well as habitat. Cryobanking offers an opportunity to preserve genetic diversity and is increasingly used for vertebrate conservation^12–14^. Cryopreservation is a method in which biological materials such as cells, tissues, organelles, or even organisms are frozen to very low temperatures, usually in liquified nitrogen for either short- or long-term storage. Survival post cryopreservation relies on two major factors; reducing the amount of ice nucleating events and reducing osmotic shock from the increase of solute concentration in the system. These can be prevented by the addition of a cryoprotectant agent (CPA) which will reduce the amount of ice crystal formation in the system, as well as a proper cooling rate, which controls the rate of transport of water across cell membranes. The cooling rate contributes to the reduction of intracellular ice formation as well as manage the solute concentration.

Cryopreservation efforts in insects have focused on economically important species such as the dipteran pests, including the screwworm, blow fly, tephritid fruit flies, etc.^15–18^ as well as agriculturally important bumblebees and honey bees^19,20^. Little effort has been placed towards the species of conservation concern. For instance, the four Lepidopterans that have been cryopreserved are agricultural pests^21–24^. Furthermore, the reported Lepidopteran procedures have been developed specifically for cryopreservation of the embryos. Embryonic cryopreservation is not practical for monarchs and many other butterfly species which requires dozens of embryos in a specific developmental stage to be treated at the same time to increase the recovery rate^25–28^. Monarchs do not lay eggs in large enough numbers for cryopreservation. A monarch female lays one egg one at a time on spaced and appropriate substrates to reduce competition as well as predation^29^. To overcome these obstacles with respect to embryonic cryopreservation, this project aimed to develop a cryopreservation protocol for the monarch butterfly spermatozoa that could be utilized for artificial insemination. An insemination technique has been reported in a species within the same order^30^. Takemura et al. (2000) developed a protocol for cryopreserving semen of the silk moth, *Bombyx mori*. The procedure reported for *Bombyx* did not assess spermatozoa viability or quality and essentially adopted a two-step uncontrolled slow-freezing protocol mimicking the procedure that is generally used for bacteria and cell cultures. Cryopreservation protocols can differ between species depending on various physical and physiological aspects, and hence it is important to ensure that the protocol to be used for at-risk species is carefully optimized and repeatable.

Here, we report the first known cryopreservation protocol for the seminal content from the male monarch butterfly that preserves the spermatozoa bundles resulting in highly intact and viable sperm.

## Methods

**Figure 1:**
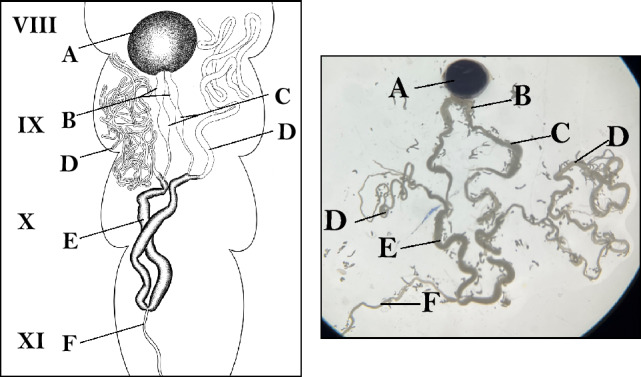
Diagram (left) and image (right) of a dissected reproductive tract from a male monarch butterfly. A—testes, B—seminal vesicle, C—accessory seminal vesicle, D—Accessory Glands, E—Ductus ejaculatorius duplex, F—Ductus ejaculatorius simplex. Roman numerals correspond to the abdominal segment in which each structure is found.

**Figure 2:**
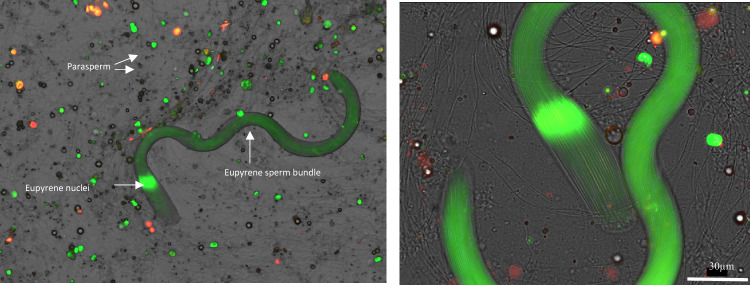
Parasperm and eusperm bundles collected from seminal vesicles and stained with SYBR-14 (green) and propidium iodide (red). (**Left image**) shows a highly stained eusperm sperm bundle in the foreground and several unstained parasperm sperm in the background. The individual eusperm bundle, live nuclei, and parasperm are highlighted in this image. (**Right image**) shows a single eusperm bundle.

Wild monarchs were collected as larvae from milkweed plants (*Asclepias syriaca* L.) in and around Fargo, North Dakota, United States, brought into the laboratory, and fed freshly collected milkweed cuttings to allow for their development to chrysalis and eclose as adults. Once adult butterflies emerged, males were separated from the females and were fed a solution of 1:5 honey:water mixture for one week to allow for gonadal maturation and sperm production. Mature males were dissected their reproductive tract was identified and removed (Fig. 1A,B). A total of 6 males (cryopreservation n = 3, control n = 3) were used in the experiment. The spermatozoa of three males were used to determine the effect of cryopreservation, and spermatozoa of three males were used as a control and their sperm was evaluated in the absence of freezing. Before males were dissected, they were very briefly exposed to −20 °C in a freezer until they were in chill coma prior to the dissection. The abdomen of the butterfly was dissected dorso-longitudinally and the gut, the fat bodies, and the tracheal strands were carefully separated from the reproductive system. The testis, which was dark red and clearly identifiable, was located in between the abdominal segments VIII and IX (Fig. 1). Posterior to the testis were the vas deferens and the seminal duplex (Fig. 1A), where the viable and mature sperm are stored until mating with the majority of the seminal contents concentrated in the duplex. The duplex was carefully excised from the body and placed in a droplet of chilled 12% Dimethyl sulfoxide (DMSO) in Grace’s insect medium (osmolality: 367 mOsm/kg; pH: 6.0) (11605094, Gibco ThermoFisher Sci., USA) in a sterile Petri plate. Grace’s insect medium is supplemented with lactalbumin hydrolysate, yeastolate, L-glutamine, and sodium bicarbonate in addition to the general components of Grace’s medium. DMSO was used as the primary cryoprotectant due to its significantly lower toxicity to insect cell types compared to other cryoprotective agents. DMSO has additional advantages such as rapid permeation during the equilibration process and egression after tissue recovery from cryopreservation. DMSO also exhibits lower metabolic interference due to its synthetic nature, unlike glycerol which plays a role in the metabolic pathways in addition to its osmotic effects^31,32^. Instead of 7–10% (1 to 1.2 M) DMSO which is generally used for spermatozoa, a 12% (~ 1.6 M) DMSO in Grace’s medium was chosen to obtain increased permeation of the cryoprotectant into the sperm bundle^33^. The duplex was then gently disrupted to release the contents into the cryoprotective medium for equilibration and cryopreservation. Due to the complex dichotomous spermatogenesis process in the lepidopterans, the contents contain two types of spermatozoa configurations—the nucleated eusperm bundles and the non-nucleated individual parasperm (Fig. 2)^34^. There are significantly more parasperm which are incapable of fertilization and their role in fertilization and reproductive physiology has only recently been characterized^35,36^. An additional complexity involves the eusperm, which are assembled into bundles containing 258 individual nucleated sperm. Prior to transfer to the female these bundles require solubilization to free the sperm cells from the bundle and to be activated^34^. Hence, it is these bundles of eusperm that were the focus of our protocol development. The cryopreservation medium containing these bundles in addition to the parasperm were loaded into half-cut (6.5 cm) 0.25 ml Cassou cryostraws (IMV Technologies, Brooklyn Park, MN, USA) preloaded with an equal volume of phosphate buffered saline (pH 7.2, 300 ± 10 mOsm/kg) (PBS). The ends of the straws were heat-sealed and transferred to a programmable rate cryogenic freezer. We employed a ramp-down freezing method. The Freeze Control® cryopreservation system (CryoLogic, Australia) was programmed with an initial ramp from 4 °C to −40 °C at a rate of −0.7 °C/minute, with a 1-min soak at −7.5 °C. The frozen straws were then quickly plunged into liquified nitrogen (LN) and then stored in the liquid phase of a storage LN Dewar for long-term storage.

The ramp-down freezing method was used to reduce the likelihood of cellular damage by reducing the probability of intracellular ice formation and osmotic shock^26,37^. Thawing and post-thaw sample processing after removal from LN storage is as critical to successful cryopreservation as the steps leading up to storage in liquid nitrogen. Warming too slowly could lead to injurious recrystallization, and longer exposure to near ambient temperatures when the toxicity of DMSO is significantly higher than at lower temperatures. Therefore, the warming rate, dilution, and rinsing of the cells to remove the cryoprotectant after cryopreservation is critical to the successful revival of the cells^33^. To revive the monarch sperm cells, the frozen samples in the cryo-straw were rapidly thawed by placing them in a 37 °C water bath. The contents of the straw (two equal volumes of semen and diluent) were quickly expelled onto a sterile petri dish and gently mixed to rapidly dilute the samples to minimize DMSO toxicity.

**Figure 3:**
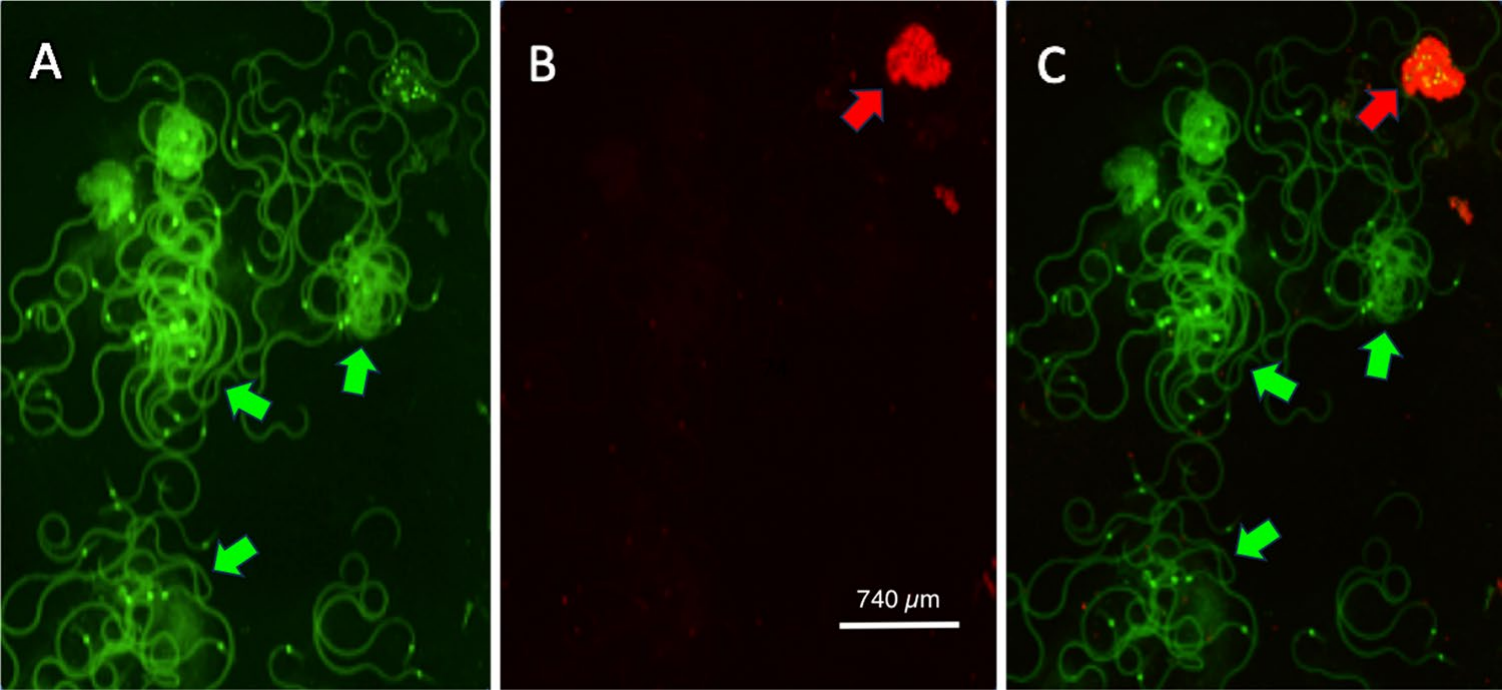
(**A**–**C**) shows cryopreserved and revived eusperm bundles from the male monarch butterfly that was stained with 100 nM of SYBR 14 and counter-stained with 15 µM of propidium iodide to differentiate between cell membrane intact cells and cell membranes compromised which is a marker for cryopreservation damage. Green arrows point to the viable eusperm bundles while the red arrow points to the non-viable testicular cells. (**A**) Shows all the nuclei stained by SYBR 14 viewed under green fluorescence. (**B**) Shows the compromised nuclei which are primarily non-spermatozoa testicular cells viewed under red fluorescence. (**C**) Shows a combination of Panel A and B viewed with both red and green fluorescence . All images have the same scale bar.

**Figure 4:**
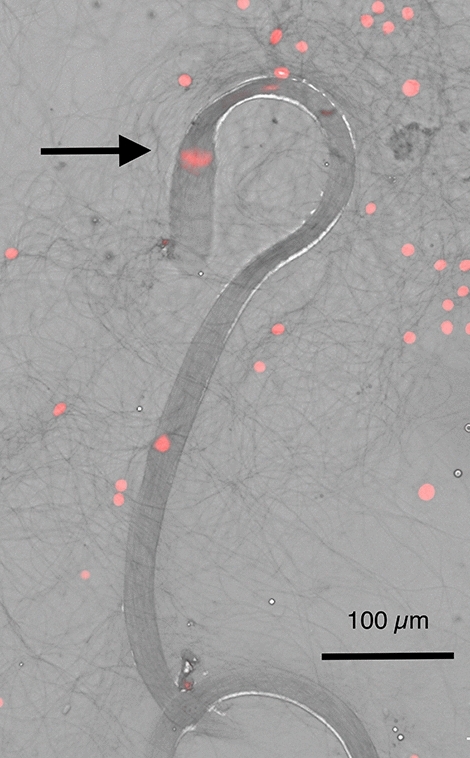
Heat-killed eusperm samples serve as negative controls. Semen smear on a glass slide was flame-heated to kill the cells and then stained with propidium iodide. The arrow points to the nuclei of the spermatozoa in the eusperm bundle that has stained red due to propidium iodide permeation. Other red spots surrounding the eusperm in the image are dead cells from the seminal vesicle.

Post cryopreservation sperm cell viability was assessed by incubating the seminal suspension in the dark in 100 nM of SYBR-14 (Invitrogen; L7011) for 10 min followed by the addition of 15 µM propidium iodide (Invitrogen; L7011) for 5 min. These dyes differentially permeate and bind the nuclei in live and dead cells. Propidium iodide is incapable of penetrating cells with intact cell membranes (Fig. 3). Samples tested included non-cryopreserved sperm collected from live adult monarchs to assess initial viability, sperm that have been retrieved from cryopreservation from adult monarchs to determine the effects of the protocol, and a negative control consisting of heat-killed sperm (n = 1) (Fig. 4) to ensure that our staining protocol was properly detecting dead sperm. After staining, eusperm bundles were visualized and captured using a live cell fluorescent imaging microscope (Lionheart FX, Agilent-Biotek, Santa Clara, CA) using fluorescent sources appropriate for each stain (Figs. 2, 3). The number of viable and dead eusperm bundles were counted in five separate fields of view to determine the proportion of alive sperm in each sample. Only fields in which all eusperm bundle nuclei were visible were counted. The nuclei of the eusperm bundle stained green was counted as 1 alive eusperm, and the nuclei of the eusperm bundle stained red (or partially red) was counted as 1 dead eusperm. The proportion of surviving sperm between cryopreserved and the non-cryopreserved controls were compared using the logistic regression with sample number included in the model as a fixed effect in R^38^. Outliers and the goodness of fit were tested for the model using the DHARMa package in R^39^.

## Results/discussion

There were no significant differences in the percent survival of eusperm in non-cryopreserved samples and the percent survival of eusperm in cryopreserved samples (logistic regression, Z = −0.223, p = 0.823). There was also no effect of individual butterflies on the percent of eusperm survival (ANOVA F = 1.606, p = 0.173). The results show that the cryopreservation protocol presented here did not have any remarkable effect on the intactness of monarch butterfly eusperm bundle both in terms of disruption of the bundle and propidium iodide uptake. Nearly none of the cell membranes of any of the sperm cells within the bundle were ruptured and stained by propidium iodide and hence, a near 100% sperm viability was observed after cryopreservation, demonstrating that the suggested sperm cryopreservation procedure for monarch butterflies is a viable method for the germplasm conservation in this species. Additional studies and developments in the field are needed, including the development of an artificial insemination protocol using cryopreserved spermatozoa mixture of both eu- and parasperm to determine the fertility of the cryopreserved semen. However, this will be complicated by the fact that during mating, male monarchs transfer the seminal content in the form of a spermatophore consisting of a mixture of eusperm, parasperm as well as accessory gland secretions to the female. This in turn is stored in the bursa copulatrix of the female and the sperm cells are released and activated as needed. Sperm activation techniques in vitro are yet to be developed and fine-tuned and therefore sperm motility was not measured in this study. However, artificial insemination with cryopreserved sperm and without spermatophore reconstitution resulting in successful hatching has been successful in another lepidopteran species^40,41^. This study may serve as a template for protocol development in monarchs.

As organizations throughout North America work to conserve the monarch butterfly through traditional means, we propose that a concurrent effort to cryopreserve genetic diversity will help maximize the likelihood of ensuring the long-term viability of this critically important species. This would include not only subsets of the eastern migratory population which overwinters in Mexico (the subject of this study) but also subsets of the western population which overwinters in California, which are also in decline^42,43^. Once populations are stabilized by traditional conservation practices, cryopreserved germplasm can be used as a resource to improve the genetic diversity of the restored population which is a method that has already been utilized in vertebrates^44^.

### Supplementary Information

Supplementary Information.

## Data Availability

All data generated or analyzed during this study are included in this published article [and its supplementary information files].
